# Isolated retroiliac ureter causing hydronephrosis in a young adult female: a case report

**DOI:** 10.1186/s13256-026-06362-8

**Published:** 2026-08-07

**Authors:** Wondwosen Mengist Dereje, Melkie Setotaw Demlaw, Desalegn Kefale Aegash, Melaku Tessema Kassie, Amare Getachew Getie

**Affiliations:** 1 College of Medicine and Health Sciences, University of Gondar, Gondar, Ethiopia; 2 Department of Surgery, College of Medicine and Health Sciences, University of Gondar, Gondar, Ethiopia

**Keywords:** Retroiliac ureter, Obstructive uropathy, Congenital anomalies, Case report

## Abstract

**Background:**

A retroiliac ureter is an exceptionally rare congenital anomaly characterized by a posterior course of the ureter relative to the iliac vessels. Its nonspecific clinical and radiological features frequently lead to misdiagnosis, and definitive diagnosis is often made only intraoperatively. The rarity of isolated cases, combined with the diagnostic challenge and delayed recognition, underscores the importance and novelty of reporting this case.

**Case presentation:**

A 20-year-old Black Ethiopian female presented with a three-year history of right flank pain that had progressively worsened over the preceding year. The pain was associated with urinary frequency, urgency, and dysuria. She had undergone multiple courses of treatment for recurrent urinary tract infections; however, her symptoms persisted despite therapy.

Given the prolonged duration of symptoms and recurrent infections, further evaluation was performed. Abdominopelvic ultrasonography and computed tomography revealed evidence of ureteral obstruction. Based on the clinical and imaging findings, surgical intervention was indicated, and the patient underwent ureteral end-to-end anastomosis with placement of a double-J (D-J) stent.

**Conclusion:**

A retroiliac ureter is a rare congenital urological anomaly that often presents with nonspecific clinical and radiologic findings, which may lead to delayed diagnosis. Failure to recognize this condition may result in progressive obstruction and renal impairment. This case highlights the importance of maintaining a high index of clinical suspicion in patients with unexplained ureteral obstruction, as timely surgical intervention can effectively relieve obstruction, preserve renal function, and lead to favorable clinical outcomes.

**Supplementary Information:**

The online version contains supplementary material available at https://doi.org/10.1186/s13256-026-06362-8.

## Background

A retroiliac ureter was first identified by Rotter during an autopsy in 1935 [1] and subsequently described in clinical practice by Dees [2] and Corbus et al. [3].

This condition remains extremely rare and has been reported only in medical literature. It is frequently identified only during surgery due to its subtle or inconclusive radiological features. Embryologically, a retroiliac ureter arises from vascular developmental anomalies, particularly the persistence of primitive vascular structures that alter the normal course of the ureter. Because patients often present with nonspecific symptoms and imaging findings are frequently inconclusive, definitive diagnosis usually requires surgical exploration.

### Case presentation

A 20-year-old Black Ethiopian female presented to our tertiary hospital with a three-year history of right flank pain, which had progressively worsened over the past year. She had visited several health centers for the same complaint but experienced no significant improvement. Throughout this period, she was repeatedly treated for urinary tract infections; however, her urinary frequency, urgency, and dysuria persisted. Due to the chronic and recurrent nature of her condition, she eventually sought further evaluation and management at our facility.

On presentation, the patient was in pain but hemodynamically stable. Her vital signs were as follows: blood pressure 110/70 mmHg, pulse rate 78 beats per minute, respiratory rate 17 breaths per minute, temperature 36.2 °C, and oxygen saturation 97% on room air.

All systemic physical examinations, including abdominal and genitourinary assessments, were unremarkable.

Given her longstanding symptoms, an abdominopelvic ultrasound was performed. The scan revealed moderate hydronephrosis of the right kidney, most likely secondary to an obstructive process.

**Fig. 1:**
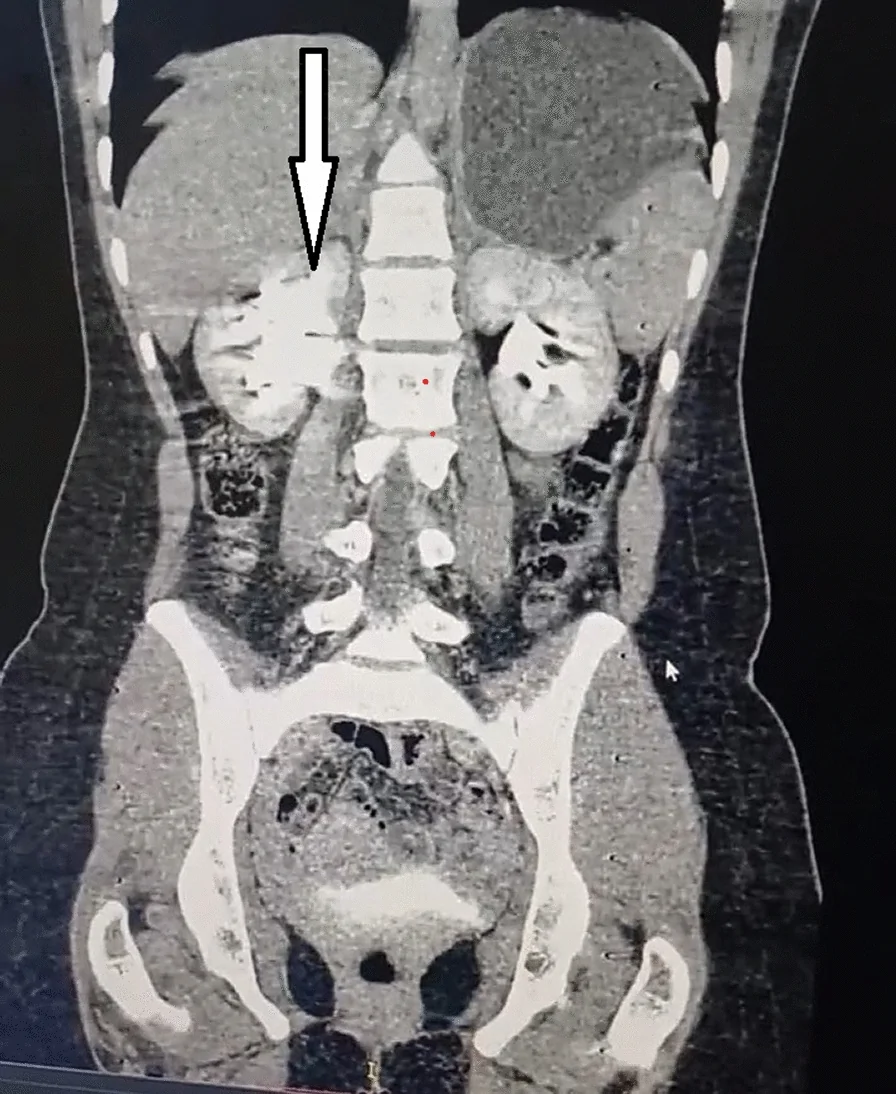
Nephrographic phase of computed tomography urography demonstrating right-sided hydronephrosis (arrow) with preserved and normal-appearing renal parenchyma

**Fig. 2:**
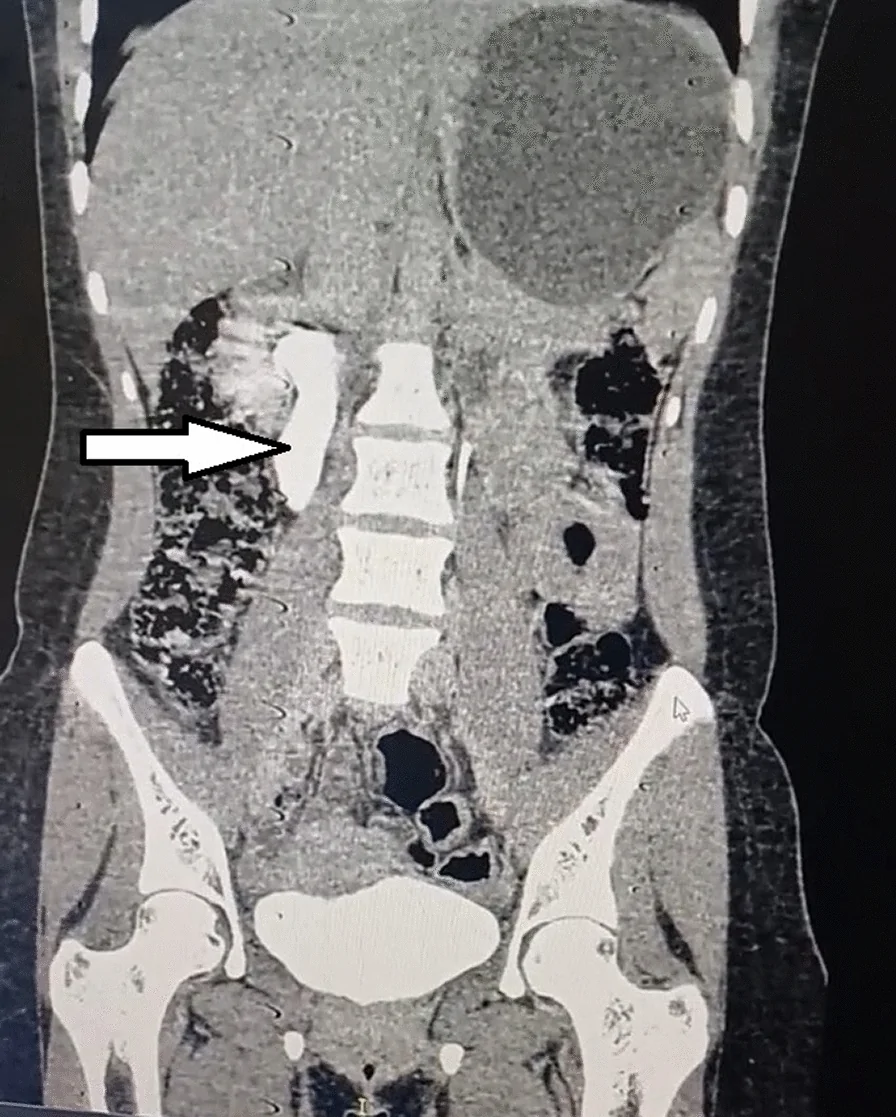
Excretory phase of computed tomography urography demonstrating proximal hydroureter (arrow) with contrast outlining the dilated ureter

For a more detailed evaluation, a CT scan with CT urography was performed. CT urography confirmed moderate hydronephrosis of the right kidney (Fig. 1), with a parenchymal thickness of 22 mm and dilation of the proximal right ureter (Fig. 2). Approximately 6.5 cm distal to the right pelviureteric junction (PUJ), a segment of tapered narrowing was observed, without evidence of renal or ureteric calculi. Additionally, the right pelvicalyceal system was distended and opacified with contrast extending into the proximal ureter, with delayed contrast passage at the level of the narrowing during the urographic phase. These findings were consistent with right moderate hydronephrosis and proximal hydroureter secondary to a proximal ureteric stricture.

**Fig. 3:**
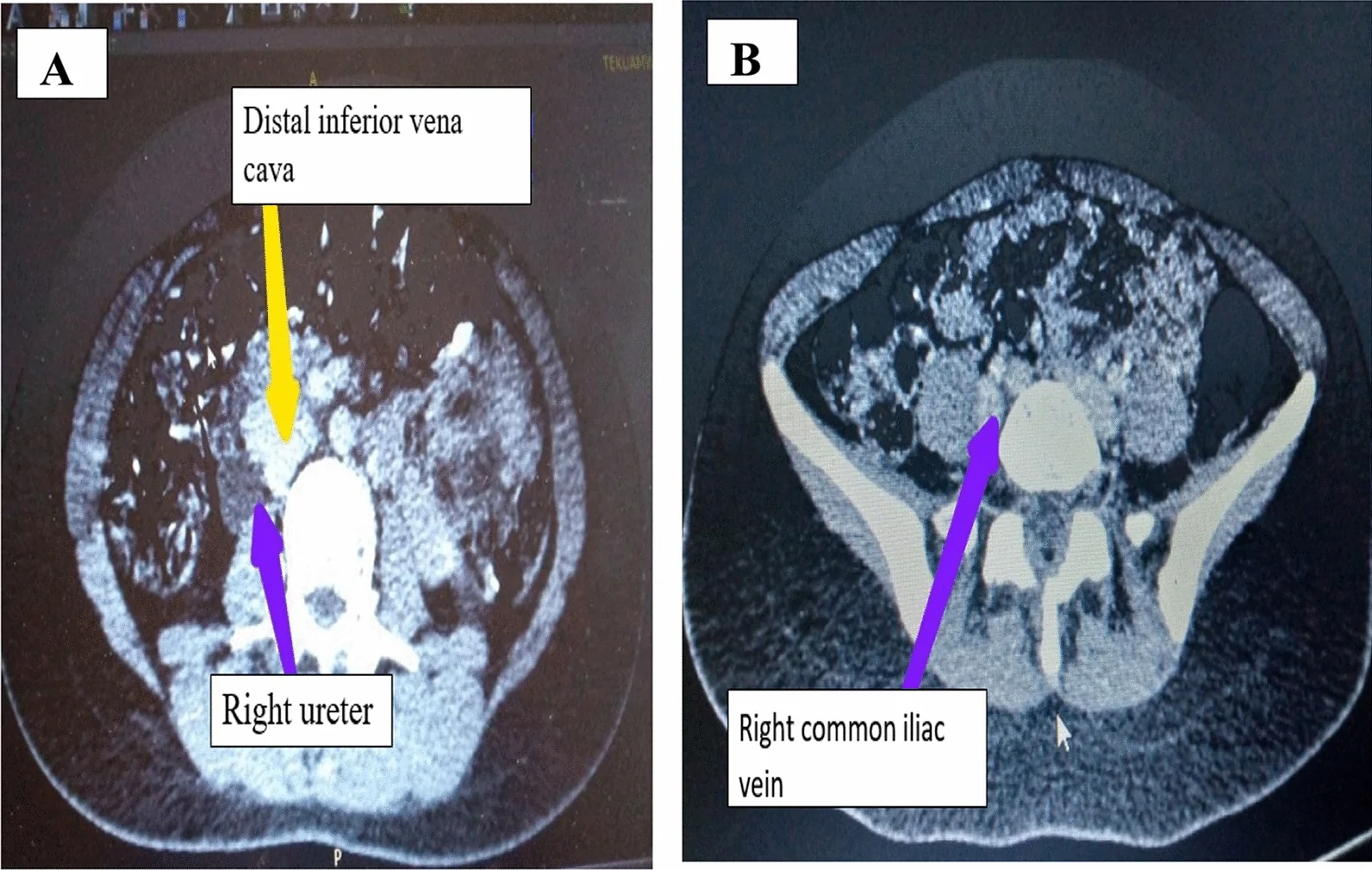
**A** and **B** Axial computed tomography urography images demonstrating the distal inferior vena cava and right ureter (Fig. 3A), as well as the right common iliac vein (Fig. 3B)

Figure 3A and B demonstrates axial CT urography images illustrating the anatomical relationships of the distal inferior vena cava, right ureter, and right common iliac vein.

Based on the patient’s clinical presentation and imaging findings, a diagnosis of retrocaval ureter was initially considered, as the CT scan demonstrated mid-pelvic ureteral obstruction. Given that retrocaval ureter is more commonly reported than retroiliac ureter, it was considered the most likely diagnosis and this diagnosis was prioritized. The findings were subsequently discussed with the patient and her family.

Additionally, the patient underwent laboratory investigations, which included a complete blood count showing white blood cells (WBC) 6.4 × 10^9^/L, hemoglobin 134 g/L, hematocrit 0.44 L/L, and platelets 359 × 10^9^/L. Urinalysis revealed a pH of 6, specific gravity 1.030, moderate epithelial cells, 3 RBCs/HPF, and 2 WBCs/HPF. Renal function tests were within normal limits, with blood urea nitrogen (BUN) 6.4 mmol/L and serum creatinine 41 µmol/L. Serum electrolytes were also normal: sodium 139 mmol/L, potassium 4.15 mmol/L, calcium 1.24 mmol/L, and chloride 105 mmol/L. Urine culture showed no bacterial growth.

**Fig. 4:**
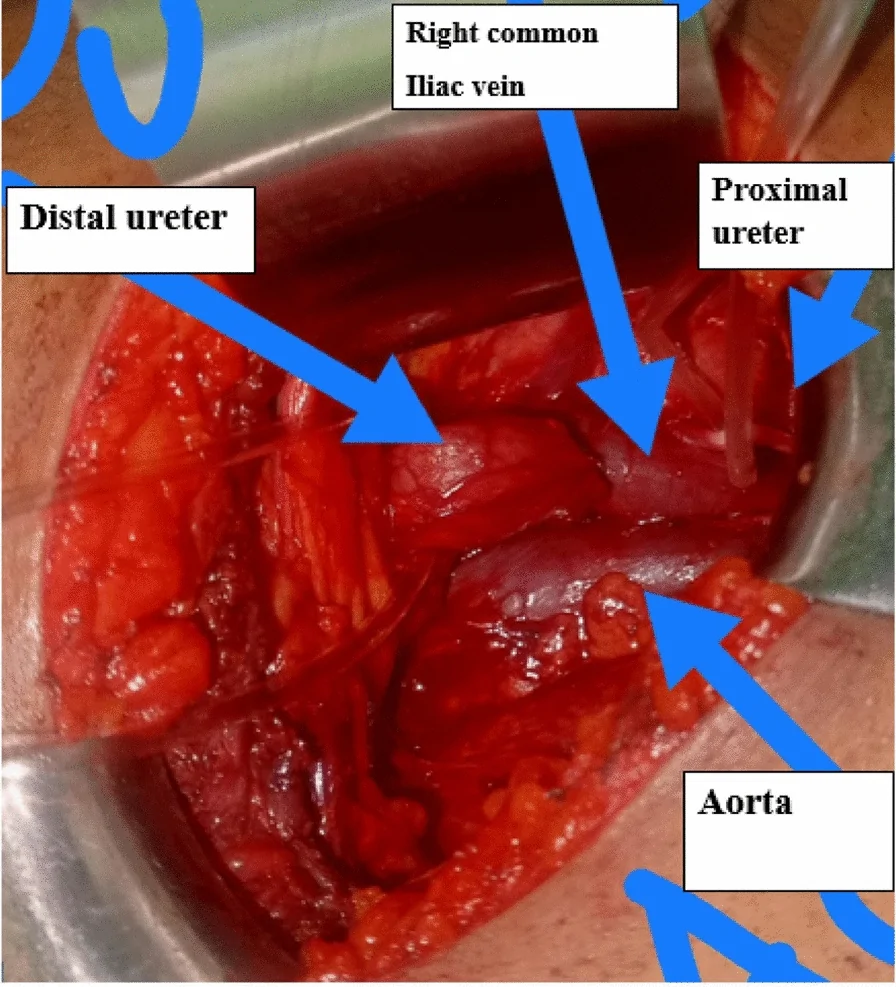
Intraoperative view demonstrating the right ureter, showing both its proximal and distal segments coursing posterior to the right common iliac vein

Based on her clinical presentation and imaging findings, surgical management was recommended. After informed consent was obtained from the patient and her family, she was taken to the operating room. Because minimally invasive surgical equipment was unavailable in the clinical setting, an open surgical approach was the only feasible option. Under general anesthesia, a right subcostal flank incision was made. The paranephric fat was dissected to expose the Gerota fascia, which was then opened anteriorly. The ureter was identified, and a narrowed ureteral segment was identified. The right ureter was found to pass posterior to the iliac vessels, confirming the diagnosis of a retroiliac ureter (Fig. 4, Video 1). A thorough examination for additional anomalies was performed, and none were identified.

The ureter was transected at the narrowed segment. Ureteral repositioning was performed, followed by uretero-ureterostomy with placement of a double-J (D-J) stent.

Following the completion of the end-to-end ureteral anastomosis and placement of the double-J stent, the patient was transferred to the post-anesthesia care unit (PACU). After 12 hours of monitoring in the PACU, she was moved to the ward, where she remained for three days before being discharged home with scheduled outpatient follow-up at the urology clinic.

At her two- and four-week outpatient follow-ups, the patient reported no new complaints and demonstrated marked improvement in her previous urinary symptoms. At the fourth-week follow-up, an ultrasound showed significant resolution of hydronephrosis, and renal function testing revealed a serum creatinine of 37 µmol/L. The ureteral stent was removed without complications. The 4-week follow-up visit was her final visit to our facility, as we were informed that she would be relocating to another city with her family.

Upon discharge from our institution, a referral letter and complete medical records were provided to facilitate continued care at her new location, and the family was advised to maintain regular follow-up. We also assured them of our availability for further communication if needed.

Follow-up information was obtained through two communications with the patient's family and treating physicians. Most recently, communication with her physician one year after surgery confirmed complete resolution of symptoms and normal renal function.

Although we were unable to obtain follow-up imaging from the hospital where she is being monitored, the physician has informed us that there has been a complete resolution of the hydronephrosis.

## Discussion

Congenital abnormalities of the kidney and urinary tract (CAKUT) encompass a broad spectrum of structural malformations that occur during the development of the kidneys and urinary tract. CAKUT accounts for approximately 20% of all birth defects and represents a significant cause of pediatric end-stage renal disease [4].

Under normal conditions, the ureter measures approximately 25–30 cm in length, extending from the renal pelvis to the bladder, with an internal diameter of 3–5 mm along its course, which may transiently dilate during peristalsis. Three normal anatomical narrowings are present at the ureteropelvic junction, pelvic brim, and ureterovesical junction [5, 6].

A retroiliac ureter is an exceedingly rare urological anomaly in which the ureter passes posterior to, or deep beneath, the iliac vessels, deviating from its usual anatomical pathway [5].

This anomaly is believed to originate from a vascular developmental defect, and patients often present with associated vascular or urogenital anomalies. Furthermore, patients with this anomaly are frequently diagnosed at an early age, as they commonly experience recurrent urinary tract infections and associated anomalies [6]. By the fourth week of gestation, the ventral root of the umbilical artery normally regresses, while the dorsal intersegmental branch connects the fused aorta and umbilical artery to form the common iliac artery. In cases where the ventral root persists and the dorsal root fails to develop, the ureter may become posteriorly entrapped, resulting in a retroiliac course [7].

In the reported case, the patient had an isolated retroiliac ureter with no associated congenital anomalies of the renal or other systems, making this presentation extremely rare. In this patient, the diagnosis was established at a later stage; despite recurrent urinary tract infections, the possibility of the current diagnosis had not been previously considered.

Retrocaval ureter, also referred to as preureteral vena cava or circumcaval ureter, is a rare congenital anomaly in which the ureter courses posterior to the inferior vena cava (IVC) as a result of abnormal IVC development [8, 9]. In this condition, the proximal ureter passes behind the IVC, typically at the level of the third lumbar vertebra [9]. This anomaly results from persistence of the right subcardinal vein as the renal segment of the IVC, rather than the right posterior cardinal vein, which normally lies lateral to the ureter during weeks 4–8 of intrauterine development [10].

The prevalence of retrocaval ureter is estimated to range from approximately 1 in 1000 [11] to 1 in 1100 live births [12]. In contrast, retroiliac ureter has been reported only in a limited number of case reports, making retrocaval ureter the more common anomaly. In the present case, retrocaval ureter was initially considered because of its higher reported prevalence compared with retroiliac ureter.

Clinically, patients with retrocaval ureter may present with nonspecific symptoms similar to those seen in retroiliac ureter, including recurrent urinary tract infections and flank pain. Retroiliac ureter remains particularly challenging to diagnose preoperatively due to its nonspecific radiologic features and is often identified intraoperatively during surgery for presumed mid-ureteric obstruction [1, 2].

Imaging studies may demonstrate hydroureteronephrosis extending to the L4–L5 level, a feature that can help differentiate retroiliac ureter from retrocaval ureter, in which ureteral dilatation typically occurs above the L3 level [5]. Despite this, accurate preoperative diagnosis of retroiliac ureter based on imaging alone remains challenging. Radiologic findings such as ureteral kinking, obstruction at the sacroiliac level, or ureteral tortuosity should raise clinical suspicion. Intraoperative assessment continues to be the gold standard for definitive diagnosis and management [5, 13].

In cases of retrocaval ureter, several diagnostic modalities may be employed; however, current practice favors less invasive and more precise imaging techniques, including computed tomography (CT) and magnetic resonance imaging (MRI). Spiral CT, in particular, has become the preferred modality for diagnosing retrocaval ureter because of its ability to clearly delineate both the ureter and the inferior vena cava [14, 15].

Although MRI was not available in our setting, CT urography was performed and demonstrated hydronephrosis and hydroureter. While retroiliac ureter has been diagnosed preoperatively using advanced imaging techniques, radiologist experience plays a crucial role, as the diagnosis in our patient was ultimately established intraoperatively.

Functional assessment of the kidneys can be performed to determine whether the kidney on the side of the obstruction is functioning normally. Various study methods have been described for this purpose, including comparisons among different techniques. CT scan with volumetry and nuclear renography are the most commonly used modalities.

Functional assessment is performed using radionuclide studies, with dynamic renography employed to evaluate differential renal function and urinary drainage. Static technetium-99m dimercaptosuccinic acid (Tc-99m DMSA) scintigraphy is used to assess the renal parenchyma. Catheter cystography techniques have evolved, allowing a marked reduction in radiation exposure through the use of isotopes (direct radioisotope cystography, DIC) or, more recently, ultrasound cystography. Additionally, cystography can be performed without bladder catheterization using indirect radioisotope cystography (IRC), which offers a completely noninvasive and physiological approach while providing additional diagnostic information [16].

Nuclear renography is most commonly performed using Tc-99 m mercaptoacetyltriglycine (MAG3) and Tc-99 m diethylenetriaminepentaacetic acid (DTPA). Other radiotracers include Tc-99 m sestamibi, Ga-68 ethylenediaminetetraacetic acid (EDTA), and F-18 fluorodeoxyglucose (FDG) [17–19].

CT-based volume measurements may be more accurate than nuclear scans, as they are less influenced by factors such as body habitus, kidney position, and operator variability. Renal volume has also been suggested to be a more sensitive indicator of renal mass than any single linear measurement [20].

Many centers utilize multidetector computed tomography with three-dimensional reconstruction (MDCT) to evaluate renal size and vascular anatomy. A key advantage of this technique is that the volume of each kidney can be readily calculated using 3D reconstruction [21, 22]. Several studies have demonstrated a correlation between renal volumes calculated by MDCT and nephron mass [21, 23].

In the reported case, assessment of renal function was warranted given the patient’s prolonged urinary tract infections attributed to the obstruction; however, due to resource limitations in the clinical setting, functional assessment could not be performed.

Nguyen et al. reviewed 24 cases of retroiliac ureter spanning a wide age range of 3–70 years. The review reported an equal male-to-female distribution, with a predominance of right-sided anomalies. Patients commonly presented with recurrent urinary tract infections, flank pain, lower abdominal discomfort, and hypertension. In some cases, the condition was diagnosed incidentally during unrelated surgical procedures or at autopsy [5].

Known risk factors for CAKUT include intrauterine exposures, such as maternal use of angiotensin converting enzyme (ACE) inhibitors, as well as a positive family history of urinary tract malformations [24].

Management of retroiliac ureter primarily involves surgical correction, including ureteral transposition, uretero-ureterostomy, or ureteroneocystostomy, depending on the specific anatomical considerations. In advanced cases where renal function is severely compromised, nephrectomy may be necessary to prevent further complications [25, 26]. In the reported case, despite the patient presenting after a prolonged period of recurrent urinary tract infections and the lack of functional renal assessment modalities in the clinical setting, timely ureteral transposition and uretero-ureterostomy were performed, resulting in clinical improvement.

## Conclusion

A retroiliac ureter is an exceedingly rare congenital anomaly of the urinary tract that can lead to ureteric obstruction and hydronephrosis. It is associated with abnormal vascular development during embryogenesis, resulting in posterior passage of the ureter relative to the iliac vessels. Most reported cases are associated with other renal or vascular anomalies, and patients typically present with nonspecific symptoms such as flank pain and recurrent urinary tract infections. Diagnosis can be challenging due to subtle imaging findings and may be confirmed intraoperatively, with surgical correction remaining the mainstay of management. The present case is particularly rare, as it occurred without identifiable risk factors or associated anomalies, highlighting the importance of recognizing and reporting such unusual presentations.

## Limitation

Although this case represents a rare occurrence in clinical practice, there were limitations, primarily due to resource constraints. Preoperative functional assessment could not be performed because of these limitations.

Postoperative imaging should also have been performed; although an ultrasound was done during stent removal, a CT scan was not possible at the time due to equipment malfunction. She later underwent CT imaging at her new hospital, which, according to her physician, demonstrated resolution of the hydronephrosis and normal renal parenchyma. Unfortunately, we were unable to obtain the CT images for inclusion in this manuscript.

## Supplementary Information

Supplementary Material 1. Video 1: Intraoperative footage showing the right ureter passing posterior to the right common iliac vein.

## Data Availability

The datasets used and/or analyzed during the current study are available from the corresponding author on reasonable request.

## Abbreviations

CAKUT: Congenital abnormalities of the kidney and urinary tract
D-J stent: Double-J stent
CT scan: Computed tomography scan
PACU: Post anesthesia care unit
WBC: White blood cells
RBC: Red blood cells
HPF: High power field
ACE inhibitors: Angiotensin converting enzyme inhibitors
